# Supplementary material for: A prospective matched case-control study on the genomic epidemiology of colistin-resistant Enterobacterales from Dutch patients
Source: Commun Med (Lond). 2022 May 20;2:55. doi: 10.1038/s43856-022-00115-6 (PMC9122983; doi:10.1038/s43856-022-00115-6)
Supplement: Supplementary file 6 — Reporting summary [file 43856_2022_115_MOESM6_ESM.pdf]

nature research

Corresponding author(s): Karuna Vendrik

Last updated by author(s): March 2, 2022

Reporting Summary

Nature Research wishes to improve the reproducibility of the work that we publish. This form provides structure for consistency and transparency in reporting. For further information on Nature Research policies, see our Editorial Policies and the Editorial Policy Checklist.

Statistics

For all statistical analyses, confirm that the following items are present in the figure legend, table legend, main text, or Methods section.

n/a	Confirmed	
☐	☒	The exact sample size (n) for each experimental group/condition, given as a discrete number and unit of measurement
☐	☒	A statement on whether measurements were taken from distinct samples or whether the same sample was measured repeatedly
☐	☒	The statistical test(s) used AND whether they are one- or two-sided<br>*Only common tests should be described solely by name; describe more complex techniques in the Methods section.*
☐	☒	A description of all covariates tested
☐	☒	A description of any assumptions or corrections, such as tests of normality and adjustment for multiple comparisons
☐	☒	A full description of the statistical parameters including central tendency (e.g. means) or other basic estimates (e.g. regression coefficient) AND variation (e.g. standard deviation) or associated estimates of uncertainty (e.g. confidence intervals)
☐	☒	For null hypothesis testing, the test statistic (e.g. F, t, r) with confidence intervals, effect sizes, degrees of freedom and P value noted<br>*Give P values as exact values whenever suitable.*
☒	☐	For Bayesian analysis, information on the choice of priors and Markov chain Monte Carlo settings
☒	☐	For hierarchical and complex designs, identification of the appropriate level for tests and full reporting of outcomes
☐	☒	Estimates of effect sizes (e.g. Cohen's d, Pearson's r), indicating how they were calculated

Our web collection on statistics for biologists contains articles on many of the points above.

Software and code

Policy information about availability of computer code

Data collection	No commercial software was used.
Data analysis	STATA SE version 15.1

For manuscripts utilizing custom algorithms or software that are central to the research but not yet described in published literature, software must be made available to editors and reviewers. We strongly encourage code deposition in a community repository (e.g. GitHub). See the Nature Research guidelines for submitting code & software for further information.

Data

Policy information about availability of data

All manuscripts must include a data availability statement. This statement should provide the following information, where applicable:

- Accession codes, unique identifiers, or web links for publicly available datasets
- A list of figures that have associated raw data
- A description of any restrictions on data availability

Raw NGS sequence data of all sequenced isolates were deposited in the Sequence Read Archive and plasmids with mcr genes in GenBank of the National Centre for Biotechnology Information (NCBI) under BioProject ID PRJNA754858. Microbiological characteristics of all sequenced isolates are shown in Supplementary Data 2 and 3. The epidemiological datasets generated and/or analysed during the current study are available from the corresponding author on reasonable request. Laboratory and hospital data from questionnaires included in this study and from ISIS-AR are available from the corresponding author upon reasonable request and with permission of the participating laboratories.

Field-specific reporting

Please select the one below that is the best fit for your research. If you are not sure, read the appropriate sections before making your selection.

☒ Life sciences ☐ Behavioural & social sciences ☐ Ecological, evolutionary & environmental sciences

For a reference copy of the document with all sections, see nature.com/documents/nr-reporting-summary-flat.pdf

Life sciences study design

All studies must disclose on these points even when the disclosure is negative.

Sample size	No sample size was calculated. Laboratories were invited to send all colistin-resistant Escherichia coli or Klebsiella pneumoniae (COLR-EK) isolates until a maximum of five and matching colistin-susceptible isolates (COLS-EK), matched on patient location, material of origin and bacterial species. After confirmatory tests, 72 COLR-EK/COLS-EK pairs were included. The incidence rate of patients with COLR-EK isolates was one of the outcomes of the study.
Data exclusions	No data were excluded from the analyses.
Replication	This study is set up with strict inclusion criteria and a highly structured protocol. Experimental data were generated using well established, reproducible, robust and validated methodology, including next-generation sequencing. Where applicable, positive and negative controls were included and these showed highly reproducible results.
Randomization	This is a matched case-control study and therefore randomization was not applicable. Covariates were controlled for by multivariate logistic regression.
Blinding	There was no blinding in this matched case-control study. Persons that filled in the questionnaire were not blinded for colistin susceptibility testing results. This was not possible, because they were asked to fill in the questionnaire at the moment that the patient was included in the study and each first included patient had a colistin-resistant isolate and the same persons were asked to send the first following colistin-susceptible isolate. The researcher that analysed the data was also not blinded for colistin-susceptibility testing results.

Reporting for specific materials, systems and methods

We require information from authors about some types of materials, experimental systems and methods used in many studies. Here, indicate whether each material, system or method listed is relevant to your study. If you are not sure if a list item applies to your research, read the appropriate section before selecting a response.

Materials & experimental systems

n/a	Involved in the study	
☒	☐	Antibodies
☒	☐	Eukaryotic cell lines
☒	☐	Palaeontology and archaeology
☒	☐	Animals and other organisms
☒	☐	Human research participants
☒	☐	Clinical data
☒	☐	Dual use research of concern

Methods

n/a	Involved in the study	
☒	☐	ChIP-seq
☒	☐	Flow cytometry
☒	☐	MRI-based neuroimaging
