# Supplementary material for: A prospective matched case-control study on the genomic epidemiology of colistin-resistant Enterobacterales from Dutch patients
Source: Commun Med (Lond). 2022 May 20;2:55. doi: 10.1038/s43856-022-00115-6 (PMC9122983; doi:10.1038/s43856-022-00115-6)
Supplement: Supplementary file 8 — Description of Additional Supplementary Files [file 43856_2022_115_MOESM8_ESM.docx]

**Description of Additional Supplementary Files**

**File Name:** Supplementary Data 1

**Title:** Characteristics of patients carrying COLR-EK or COLS-EK isolates.

**Legend:** ¤ N total= biologically independent patients with data available per variable. * Resembling the incidence rate ratio. ~ Wilcoxon signed rank test: no significant difference. ^#^ p<0.05 in matching-adjusted logistic regression with correction for matched variables. ^ Egypt and unknown country. ^^ Sweden and Austria. ^$^ Ghana. ^$$^ all selective intestinal/oropharyngeal decontamination with colistin. ^§^ the total number of patients with known data is not certain, because there was no ‘unknown’ option in the questionnaire for this variable. Abbreviations: COLR-EK: colistin-resistant *E. coli* or *K. pneumoniae*, COLS-EK: colistin-susceptible *E. coli* or *K. pneumoniae*, IQR: interquartile range.

**File Name:** Supplementary Data 2

**Title:** Microbiological characteristics of the 66 sequenced *E. coli* isolates.

**Legend:** Abbreviations: MIC: minimal inhibitory concentration, MLST: multilocus sequence typing, ST: sequence type

**File Name:** Supplementary Data 3

**Title:** Microbiological characteristics of the 26 sequenced *K. pneumoniae* isolates.

**Legend:** Abbreviations: MIC: minimal inhibitory concentration, MLST: multilocus sequence typing, ST: sequence type

**File Name:** Supplementary Data 4

**Title:** Molecular characterisation of seven isolates with *mcr* genes.

**Legend:** Contig IDs, including IDs from chromosomes that start with a ‘c’ and IDs from plasmids that start with a ‘p’ are depicted on the Y-axis, with numbers after the underscore indicating the plasmid numbers (_1, _2, _3, etc), and antimicrobial resistance genes with antibiotic classes are depicted on the X-axis. The presence of antimicrobial resistance genes is indicated with black boxes. Striped boxes indicate that only next-generation sequencing (NGS) data is available or that a gene was only found with NGS. Abbreviations: bp: base pairs, LPS: lipopolysaccharide, mdf(A): multidrug resistance, MLST: multilocus sequence typing.

**File Name:** Supplementary Data 5

**Title:** Overview of isolates with *mcr* genes or chromosomal mutations, potentially involved in colistin resistance.

**Legend:** Abbreviations: bp: base pairs, MIC: minimal inhibitory concentration.
